# A Graphene Oxide-Based Fluorescent Platform for Probing of Phosphatase Activity

**DOI:** 10.3390/nano6010020

**Published:** 2016-01-18

**Authors:** Ting Sun, Ning Xia, Lin Liu

**Affiliations:** College of Chemistry and Chemical Engineering, Anyang Normal University, Anyang 455000, China; zhaofeng@aynu.edu.cn

**Keywords:** graphene oxide, fluorescent biosensors, phosphatase, peptide substrate, fluorescence quenching

## Abstract

We presented a strategy for fabricating graphene oxide (GO)-based fluorescent biosensors to monitor the change of phosphorylation state and detect phosphatase activity. By regulating the interaction between the negatively charged phosphate group and the positively charged amino residue, we found that GO showed different quenching efficiency toward the phosphorylated and dephosphorylated dye-labeled peptides. To demonstrate the application of our method, alkaline phosphatase (ALP) was tested as a model enzyme with phosphorylated fluorescein isothiocyanate (FITC)-labeled short peptide FITC–Gly–Gly–Gly–Tyr(PO_3_^2−^)–Arg as the probe. When the negatively charged phosphate group in the Tyr residue was removed from the peptide substrate by enzymatic hydrolysis, the resulting FITC–Gly–Gly–Gly–Tyr–Arg was readily adsorbed onto the GO surface through electrostatic interaction. As a result, fluorescence quenching was observed. Furthermore, the method was applied for the screening of phosphatase inhibitors.

## 1. Introduction

Phosphorylation and dephosphorylation play important functions in cellular regulation and signaling processes, such as metabolism, gene transcription and translation, cytoskeletal rearrangement, and apoptosis [1]. Numerous inhibitors have been shown to be promising drugs for regulating the process of phosphorylation/dephosphorylation [2]. Thus, a simple and sensitive method to monitor the change of phosphorylation state and detect phosphatase activity is extremely valuable for biomedical applications [3].

Graphene oxide (GO) is a novel one-atom-thick two-dimensional carbon material with excellent aqueous process ability, amphiphilicity, surface functionalizability, surface-enhanced Raman scattering (SERS) property and fluorescence quenching ability [4,5]. Based on the unique physicochemical and structural properties, GO has attracted interest among a wide variety of fields, including biosensors, electrochemical energy storage, and electronics [6,7,8,9]. Intriguingly, GO has shown extraordinarily high quenching ability toward fluorescently labeled DNA, peptides and antibodies because of the prominent nanoscale-surface energy transfer (NSET) effect from fluorophore to GO [10,11,12,13,14]. Thus, there has been widespread interest in the development of GO-based fluorescent methods for the probing of enzyme activity [15], imaging of cells and animals [16], and measuring the concentration level of various analytes, such as DNA [17,18], proteins [19], metal ions [20], ATP and other compounds [21]. Typically, the GO-based fluorescent sensors are categorized into two types. First, the target-receptor interaction induces the change in the conformation or orientation of a fluorescently labeled receptor assembled onto the GO surface, which in turn causes the release of the labeled receptor from the GO surface or the change in distance between the GO and fluorophore. Second, enzymatic digestion of a fluorescently labeled substrate pre-immobilized onto the GO surface releases the fluorophore into solution, thus resulting in an increase in the fluorescent signal. Usually, the fluorescently labeled probe can be assembled onto the GO surface by electrostatic/π-stacking interactions or covalent coupling. In contrast, the approach using noncovalent electrostatic/π-stacking interactions to adsorb probe onto the GO surface is simple and sensitive for the designing of GO-based fluorescent sensors. For example, adsorption of a fluorescently labeled peptide onto GO surface through electrostatic and π-stacking interactions can cause fluorescence quenching; after interaction with a target or cleavage by an enzyme, the labeled peptide segment is released from the GO surface into solution, resulting in an increase of the fluorescence signal [15,19,22,23,24]. In this process, the adsorption behavior of the peptide on the GO is dependent on the incorporation of positively charged amino acids (Lys, His, and Arg) and aromatic ring–containing hydrophobic amino acids (Trp, Tyr, and Phe), which contribute to the electrostatic and π-stacking interactions with negatively charged GO. Considering the chemical difference in the phosphorylation/dephosphorylation reaction, we hypothesize that the quenching efficiency of GO to the phosphorylated and dephosphorylated dye-labeled peptides could be distinguished by regulating the interaction between the negatively charged phosphate group and the positively charged amino residue; thus, the change of the phosphorylation state could be determined with GO as the quencher.

To demonstrate the concept of our method, we first investigated the quenching efficiency of GO toward the phosphorylated and dephosphorylated fluorescein isothiocyanate (FITC)-labeled short peptides, FITC–Gly–Gly–Gly–Tyr(PO_3_^2−^)–Arg (denoted as FITC–GGGYpR) and FITC–Gly–Gly–Gly–Tyr–Arg (denoted as FITC–GGGYR). The results demonstrated that GO exhibits higher fluorescence quenching efficiency to the dephosphorylated peptide. Inspired by the result, alkaline phosphatase (ALP), a common phosphatase present in all human tissues throughout the entire body, was tested as a model enzyme.

## 2. Results and Discussion

### 2.1. Detection Principle of This Method

**Figure 1:**
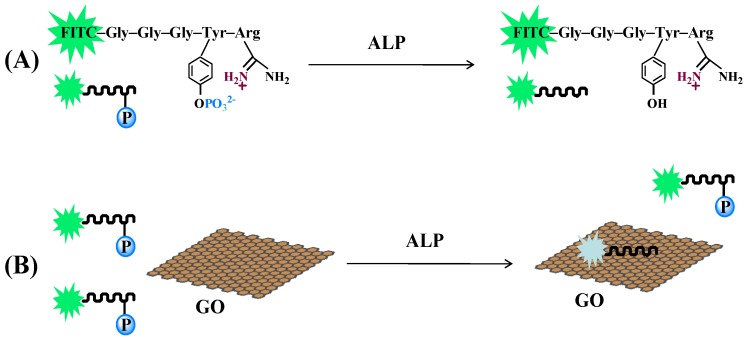
Schematic illustration of dephosphorylation process (**A**) and alkaline phosphatase (ALP) activity detection (**B**) using fluorescein isothiocyanate (FITC)–Gly–Gly–Gly–Tyr(PO_3_^2−^)–Arg (GGGYpR) and graphene oxide (GO) as the probe and the quencher, respectively.

GO is a universal quencher for diverse fluorophores due to the well-known long-range nanometal surface energy transfer [18]. It has been suggested that adsorption of a dye-labeled peptide onto the GO surface through electrostatic and π-stacking interactions between GO and amino residues in the peptide would lead to quenching of the dye fluorescence [23]. Herein, the phosphorylated dye-labeled peptide with a sequence of FITC–GGGYpR was designed and used as the ALP substrate, in which the positively charged arginine residue was included for binding of the negatively charged GO (Figure 1). The negatively charged phosphotyrosine residue could act as a molecular switch for the GO-peptide interaction. Without the addition of ALP, the negatively charged phosphate group in the Tyr residue is arranged to the positively charged guanidine group in the Arg residue, which prevents the adsorption of the peptide to the GO. On the other hand, GO will bind more weakly with the phosphorylated peptide because the negatively charged phosphate can efficiently shield the Tyr side-chain from negatively charged GO and weaken the Tyr-GO interaction. If the phosphate group was removed from the substrate by enzymatic dephosphorylation, the resulting dephosphorylated peptide FITC–GGGYR will be readily adsorbed onto the GO surface through electrostatic interaction, leading to fluorescence quenching. The change of the fluorescence intensity would depend on the amount of the resulting dephosphorylated peptide, thus enabling qualitative and quantitative measurement of the enzymatic activity of ALP.

### 2.2. Quenching Ability of GO toward Phosphorylated and Dephosphorylated Peptides

**Figure 2:**
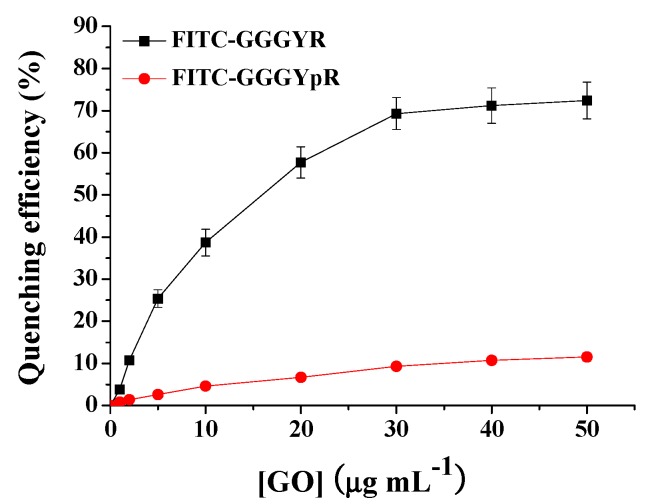
Quenching efficiency of different concentrations of GO toward phosphorylated peptide FITC–GGGYpR and dephosphorylated peptide FITC–Gly–Gly–Gly–Tyr–Arg (GGGYR).

To prove the feasibility of our method, the quenching ability of different contents of GO toward phosphorylated and dephosphorylated peptides was first investigated. The results are shown in Figure 2. It can been observed that the quenching efficiency of GO for the dephosphorylated peptide increased with the increasing GO concentration and trended to a maximal value beyond 30 μg·mL^−1^, implying that the dephosphorylated peptide had been efficiently adsorbed onto the surface of the GO. The quenching efficiency was calculated using the formula (1 − F/F_0_) × 100% to be 69.3% ± 3.8%, where F_0_ and F are the fluorescence intensities at 517 nm in the absence and presence of GO, respectively. However, only ~9.3% of the fluorescence of phosphorylated peptide was quenched by 30 μg·mL^−1^ of GO. The further increase of GO concentration did not cause higher fluorescence quenching efficiency. This result indicated that the phosphorylated peptide was not efficiently adsorbed onto the surface of the GO.

### 2.3. Feasibility for ALP Activity Assay

**Figure 3:**
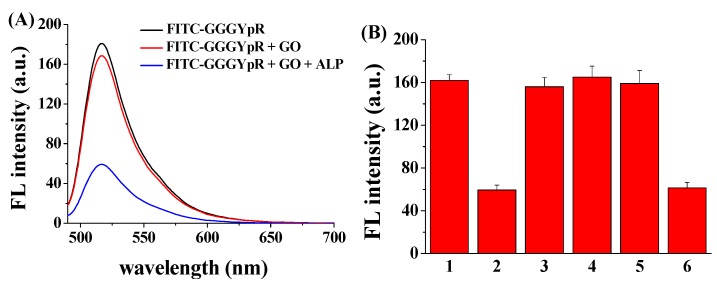
(**A**) Fluorescence spectra of FITC–GGGYpR in the presence of GO and ALP; (**B**) Fluorescence intensity of FITC–GGGYpR/GO in the absence (bar 1) and presence of ALP (bar 2), bovine serum albumin (BSA) (bar 3), lysozyme (bar 4), myoglobin (bar 5), and ALP/BSA/lysozyme/myoglobin (bar 6). The final concentrations of FITC–GGGYpR, GO, ALP, BSA, lysozyme and myoglobin are 100 nM, 50 μg·mL^−1^, 10 nM, 0.5 μg·mL^−1^, 0.5 μg·mL^−1^ and 0.5 μg·mL^−1^, respectively.

Figure 3A depicts the fluorescence spectra of FITC–GGGYpR in different systems. The fluorescence of FITC–GGGYpR decreased slightly upon the addition of the GO, indicating that FITC–GGGYpR has no (or poor) binding with GO. However, after the addition of ALP to the FITC–GGGYpR/GO mixed solution, the fluorescence intensity decreased greatly. The result indicated that the method is feasible for the probing of ALP activity. To investigate the selectivity and interference, bovine serum albumin (BSA), lysozyme and myoglobin were tested as interfering proteins. As shown in Figure 3B, the three proteins did not cause an apparent change in the fluorescence intensity. Furthermore, the addition of these proteins to the FITC–GGGYpR solution has no impact on ALP-triggered fluorescence quenching. Thus, the presence of the three proteins did not prevent the enzymatic dephosphorylation. These results supported our hypothesis that the FITC–GGGYpR can be used as a probe for analysis of the phosphatase activity.

### 2.4. Dependence on ALP Concentration and Incubation Time

**Figure 4:**
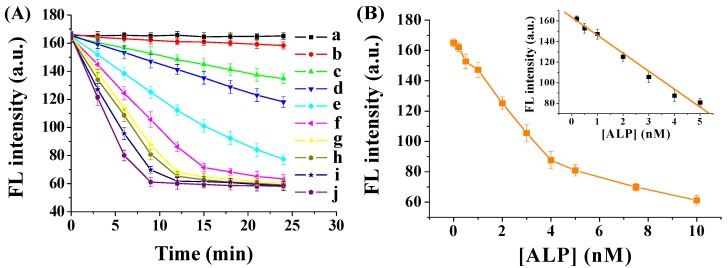
(**A**) Fluorescence intensity of FITC–GGGYpR/GO after addition of different concentrations of ALP (from a to j: 0, 0.2, 0.5, 1, 2, 3, 4, 5, 7.5 and 10 nM). The final concentrations of GO and FITC–GGGYpR are 50 μg∙mL^−1^ and 100 nM, respectively; (**B**) Dependence of fluorescence intensity on the ALP concentration.

ALP concentration and incubation time have a profound influence on the dephosphorylation reaction. Thus, we investigated the effect of ALP concentration and incubation time on the fluorescence signals. The experiments were conducted under the same condition with different amounts of ALP. The fluorescence intensity was recorded every 3 min after mixing ALP with the FITC–GGGYpR/GO solution. As shown in Figure 4A, without ALP, no apparent change was observed in the fluorescence intensity with the incubation time. The FITC–GGGYpR/GO samples mixed with ALP showed a time-dependent decrease in the fluorescence intensity. The kinetics were observed to be faster with a higher concentration of ALP, indicating that the decrease of fluorescence intensity was caused by the increased amount of dephosphorylated peptide, and that more ALP induced more dephosphorylation. Moreover, a near-linear correlation between the ALP concentration and the fluorescence intensity was observed in the range of 0.2 nM–5 nM at the 9-min incubation time (Figure 4B). The linear regression equation is expressed as F = 163.5 − 17.4 [ALP] (nM) (*R*^2^ = 0.99). The detection limit was estimated to be 0.08 nM. The relative standard deviations (RSD) are all less than 7%. These results demonstrated that the method has good sensitivity and reproducibility.

### 2.5. Determination of ALP Inhibitor

**Figure 5:**
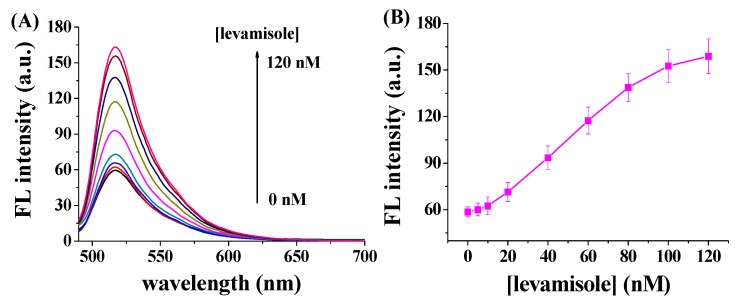
(**A**) Fluorescence spectra of 100 nM FITC–GGGYpR/GO in the presence of 10 nM ALP and different concentrations of levamisole (0, 5, 10, 20, 40, 60, 80, 100 and 120 nM); (**B**) Dependence of the fluorescence intensity on the concentration of levamisole.

The concentration level of ALP and its dephosphorylation activity have been believed to be associated with some widespread diseases, such as bone diseases, liver dysfunction, prostatic cancer and bile duct blockage [1]. For example, the concentration of ALP in normal adult is in the range of 20–140 IU/L, but obstruction of bile duct will induce the increase in the concentration of ALP. Additionally, active bone formation will cause the increase of ALP concentration. Levels are also elevated in people with untreated Celiac disease. ALP inhibitor has thus been shown to be a promising drug for curing the disease caused by ALP overexpression. We suggest that the potential ALP inhibitors could be screened by using the GO-based fluorescent method. To demonstrate the application of our method in screening the enzyme inhibitors, levamisole, a well-known ALP inhibitor, was tested with the currently optimized procedures. When FITC–GGGYpR/GO was incubated with levamisole-treated ALP, higher fluorescence signals were observed (Figure 5A). In addition, the fluorescence intensity increased significantly with increased levamisole concentrations (Figure 5B). These findings suggested that the activity of ALP was suppressed by levamisole and the inhibition was more effective at higher levamisole concentrations. From the fluorescence intensity-concentration curve, the half-maximum inhibition value (IC_50_) was found to be 53.9 nM.

## 3. Experimental Section

### 3.1. Chemicals and Materials

Fluorescein isothiocyanate (FITC)-linked short peptide (FITC–Gly–Gly–Gly–Tyr–Arg, denoted as FITC–GGGYR) and its phosphorylated form (FITC–Gly–Gly–Gly–Tyr(PO_3_^2−^)–Arg, denoted as FITC–GGGYpR) were synthesized and purified by ChinaPeptides Co., Ltd. (Shanghai, China). ALP and its inhibitor levamisole were obtained from Sangon Biotech. Co., Ltd. (Shanghai, China). Trisodiumcitrateandtris-(hydroxymethyl)aminomethane hydrochloride (Tris–HCl) was purchased from Sigma-Aldrich (Shanghai, China). GO was purchased from Nanjing XFNANO Materials Tech Co., Ltd. (Nanjing, China). GO was dissolved in deionized water solution to a final concentration of 1 mg∙mL^−^^1^ and sonicated for 1 h. The solutions of peptide and ALP were prepared with deionized water treated with a Millipore system (Simplicity Plus, Millipore Corp., Billerica, MA, USA). Before use, they were diluted to the desired concentration with Tris-HCl buffer (10 mM, pH 7.4).

### 3.2. Quenching Studies of GO toward Phosphorylated and Dephosphorylated Peptides

To investigate the fluorescence quenching ability of GO toward FITC–GGGYR and FITC–GGGYpR, 100 μL of GO suspension at a given concentration was added to 100 μL of 200 nM peptide solution (pH 7.4). After 3 min, fluorescence spectra were taken with a Varian Cary fluorescence spectrometer (Palo Alto, CA, USA) upon excitation at 470 nm. The emission wavelengths were collected with both excitation and emission slits of 5 nm.

### 3.3. Protocol for the ALP Activity Assay

For the ALP activity assay, 2 mL of 400 nM FITC–GGGYpR was first mixed with 2 mL of GO suspension. Then, 100 μL of ALP solution was added into 100 μL of the FITC–GGGYpR/GO solution for fluorescence measurement. To determine the inhibition of levamisole, ALP was pre-incubated with different concentrations of levamisole for 10 min at room temperature. Since a near-linear correlation between the ALP concentration and the fluorescence intensity was observed at the 9-min incubation time (Figure 4), the levamisole-pretreated ALP was thus allowed to react with FITC–GGGYpR/GO for 9 min. Then, the fluorescence spectra were recorded according to the same procedure described above.

## 4. Conclusions

We reported a simple GO-based fluorescent method for monitoring the change of phosphorylation state. The fluorescence quenching of the GO to the peptide was controlled by regulating the charge interaction between amino acids and GO. To demonstrate the analytical performances of this method, ALP was tested as a model enzyme. Compared with other methods, our method obviates the need for complicated instruments and is cost-effective because GO can be prepared in large quantities from graphite available at a very low cost. Furthermore, with the rational design of the substrate sequence, we believe that this concept could be useful for development of other GO-based fluorescent biosensors for enzymatic assay, such as tyrosine phosphatase and estrogen sulfatase. Additionally, the method will be valuable for the screening of new enzyme inhibitors and drugs in a high-throughput screening format using a multi-well plate reader.
